# Supplementary material for: Intrinsically weak sex chromosome drive through sequential asymmetric meiosis
Source: Sci Adv. 2025 May 7;11(19):eadv7089. doi: 10.1126/sciadv.adv7089 (PMC12057659; doi:10.1126/sciadv.adv7089)
Supplement: Supplementary file 1 — Figs. S1 to S8 Tables S1 to S8 [file sciadv.adv7089_sm.pdf]

Science Advances
AAAS

Supplementary Materials for

Intrinsically weak sex chromosome drive through sequential asymmetric meiosis

Xuefeng Meng and Yukiko M. Yamashita

Corresponding author: Xuefeng Meng, mengxf@wi.mit.edu; Yukiko M. Yamashita, yukikomy@wi.mit.edu

Sci. Adv. **11**, eadv7089 (2025)
DOI: 10.1126/sciadv.adv7089

The PDF file includes:

Figs. S1 to S8
Tables S1 to S8

Other Supplementary Material for this manuscript includes the following:

MATLAB code for the mathematical modeling

Fig. S1

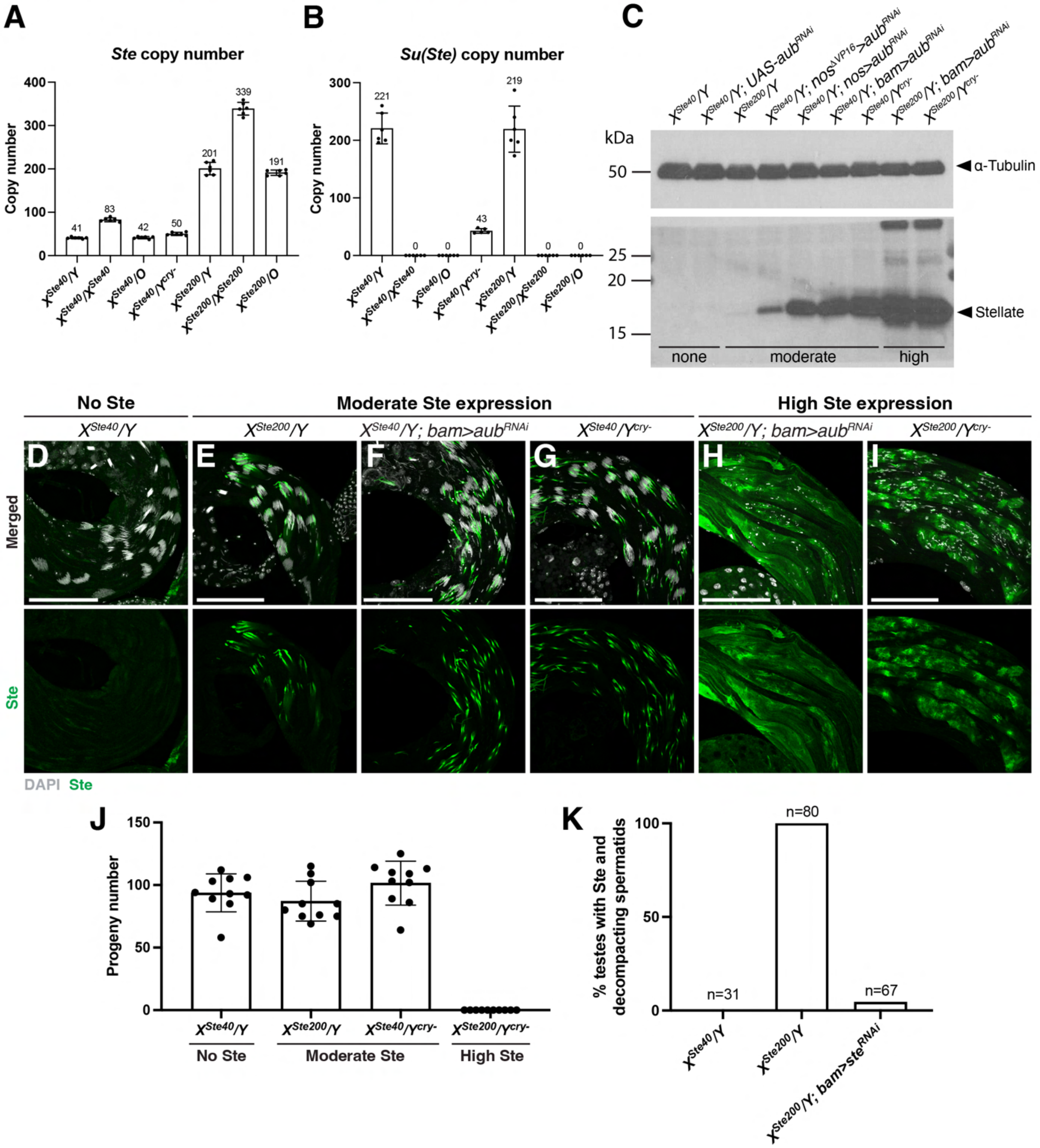

Fig. S1. Varying degrees of *Ste* derepression in different genetic backgrounds. (**A**) *Ste* copy number on the X^{Ste40} and X^{Ste200} chromosomes, determined by ddPCR. *Ste* copy number in X/Y males is half of that in X/X females. The *Ste* copy number in X/Y males is the same as that in X/O males, demonstrating that the ddPCR primers and probes are specific to *Ste* and do not detect *Su(Ste)*. (**B**) *Su(Ste)* copy number on the normal Y and Y^{cry-} chromosomes, determined by ddPCR. *Su(Ste)* is not detected in X/O males or X/X females, demonstrating that the *Su(Ste)*

ddPCR primers and probes are specific to *Su(Ste)* and do not cross-hybridize with *Ste*. (**C**) Western blotting to determine the expression level of Ste under the indicated genetic conditions, probed with anti-α-Tubulin (loading control) and anti-Ste antibodies. (**D** to **I**) Immunofluorescence staining for Ste (green) in testes from males of the indicated genotypes. Grey, DAPI. Scale bars, 100 μm. (**J**) Fertility assay for males of the indicated genotypes. Single males were crossed to two wild-type (*y w*) females for 5 days, and all progeny were counted. Ten males were assayed per genotype. (**K**) Percentage of testes expressing Ste and showing spermatid nuclear DNA compaction defects in the indicated genotypes. The number of testes scored for each genotype is shown above the bars.

Fig. S2

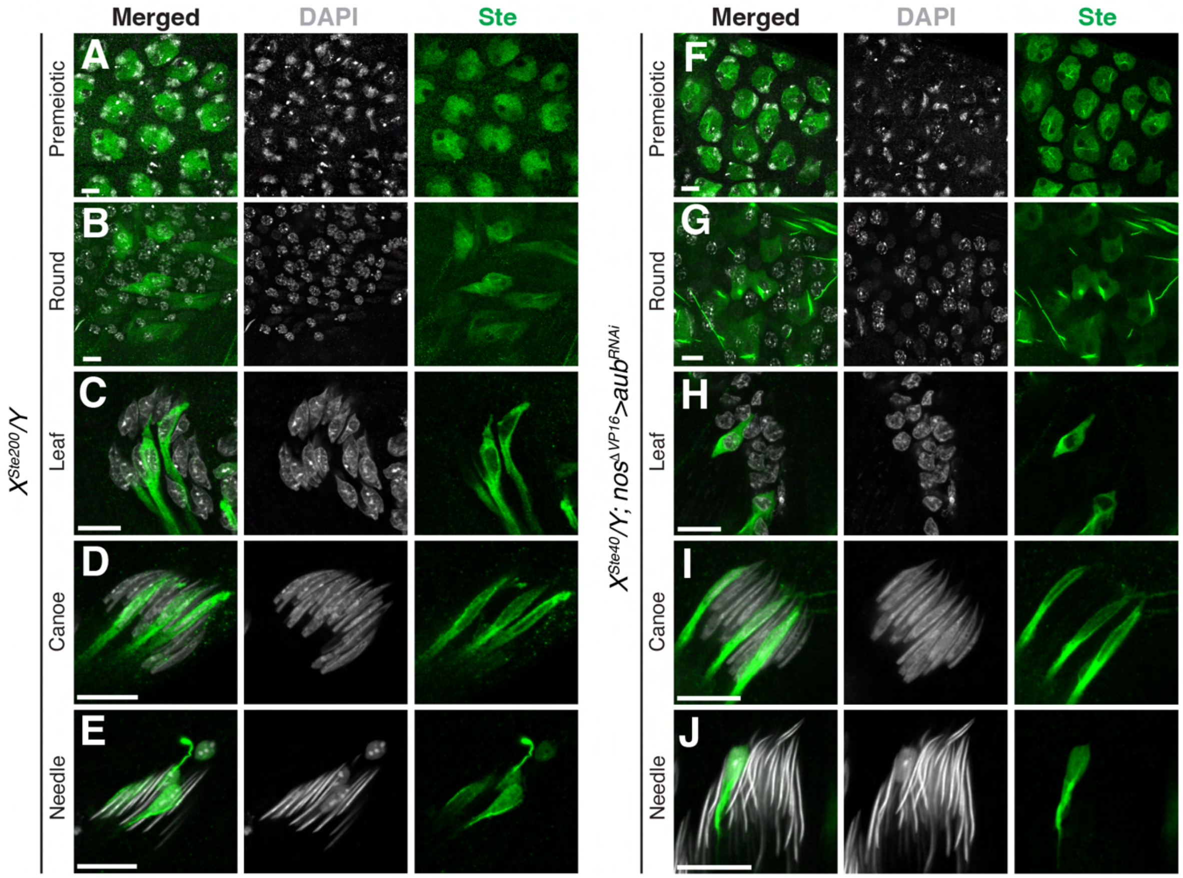

Fig. S2. Moderately expressed Ste is localized to a subset of spermatids throughout spermiogenesis. (**A** to **E**) Immunofluorescence staining for Ste (green) in the indicated germ cell developmental stages in X^{Ste200}/Y males: (A) Premeiotic spermatocytes; (B) round spermatids; (C) leaf spermatids; (D) canoe spermatids; (E) needle spermatids. Grey, DAPI. Scale bars, 10 µm. (**F** to **J**) Immunofluorescence staining for Ste (green) in the indicated germ cell developmental stages in X^{Ste40}/Y; $nos^{\Delta VP16}>aub^{RNAi}$ males: (F) Premeiotic spermatocytes; (G) round spermatids; (H) leaf spermatids; (I) canoe spermatids; (J) needle spermatids. Grey, DAPI. Scale bars, 10 µm.

Fig. S3.

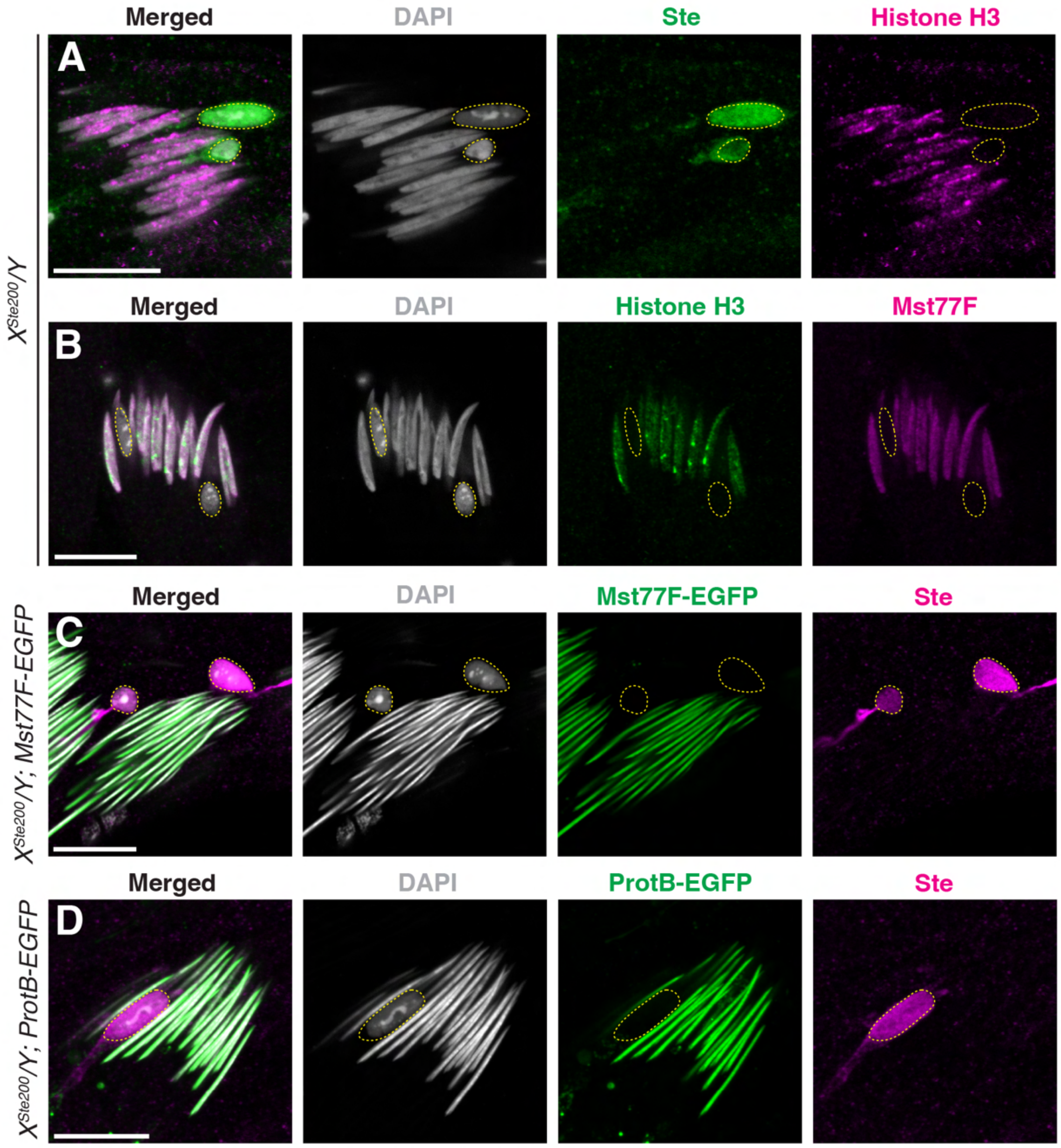

Fig. S3. Ste interferes with the histone-to-protamine transition during spermiogenesis. (**A**) Canoe-stage spermatids from X^{Ste200}/Y males stained for Ste (green), Histone H3 (magenta) and DAPI (grey), showing that Ste-containing spermatids (yellow dotted circles) do not have histones, whereas Ste-negative spermatids within the same cyst have Histone H3 signals. Scale bar: 10 µm. (**B**) Canoe-stage spermatids from X^{Ste200}/Y males stained for Histone H3 (green), Mst77F (magenta, a protamine protein) and DAPI (grey), showing that spermatids that do not have Histone H3 and fail to condense DNA (presumably Ste-containing spermatids, yellow dotted circles) also lack Mst77F. Scale bar: 10 µm. (**C**) Needle-stage spermatids from X^{Ste200}/Y; *Mst77F-EGFP* males (Mst77F-EGFP, green) stained for Ste (magenta) and DAPI (grey), showing that Ste-containing spermatids (yellow dotted circles) lack Mst77F. Scale bar, 10 µm. (**D**) Needle-stage spermatids from X^{Ste200}/Y; *ProtamineB-EGFP* males (ProtamineB-EGFP,

green) stained for Ste (magenta) and DAPI (grey), showing that Ste-containing spermatids (yellow dotted circle) lack Protamine B. Scale bar, 10 µm.

Fig. S4

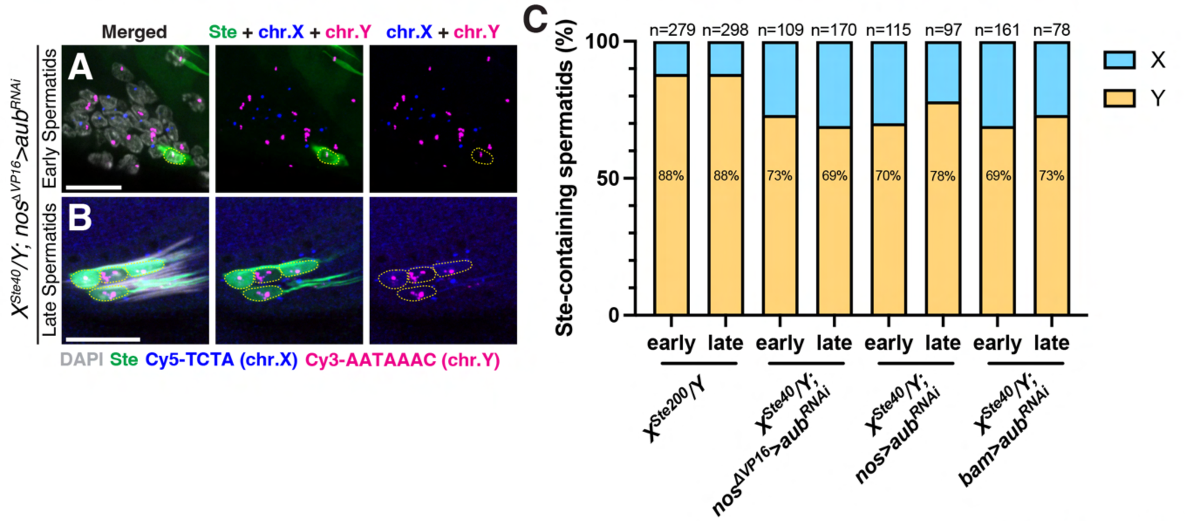

Fig. S4. Ste is preferentially localized to Y-bearing spermatids throughout spermiogenesis. (**A** and **B**) Immunofluorescence and DNA-FISH of early (leaf stage) and late (needle stage) spermatids from *X*^{Ste40}*/Y; nos*$^{\Delta VP16}$*>aub*^{RNAi} males, stained for Ste (green), Cy5-TCTA (X chromosome, blue), and Cy3-AATAAAC (Y chromosome, magenta). Nuclei of Ste-containing spermatids are indicated by yellow dotted circles, showing that they predominantly contain Y chromosomes. Scale bars, 10 µm. (**C**) Frequency of X- or Y-bearing spermatids among Ste-containing spermatids in early (round + leaf) and late (canoe + needle) stages for the indicated genotypes.

Fig. S5

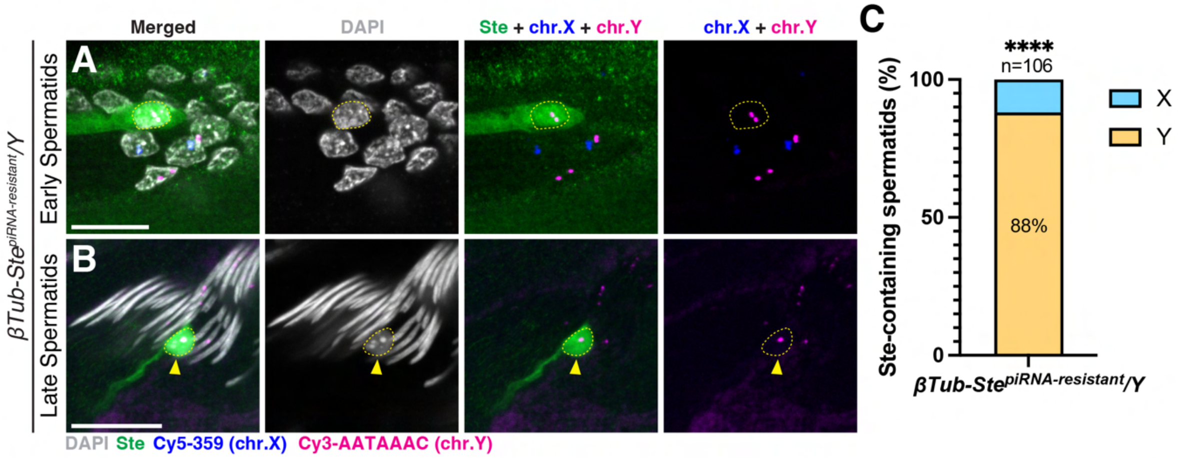

Fig. S5. Ste is sufficient to cause Y-spermatid defects. (**A** and **B**) Immunofluorescence and DNA-FISH staining of early (leaf stage) (A) and late (needle stage) (B) spermatids from males expressing the piRNA-resistant *Ste* transgene (β*Tub-Ste*$^{piRNA\text{-}resistant}$), stained for Ste (green), X chromosome (Cy5-359, blue) and Y chromosome (Cy3-AATAAAC, magenta). Yellow dotted circles indicate the nuclei of Ste-containing spermatids, which predominantly contain a Y chromosome. Yellow arrowhead indicates a Ste-containing spermatid with nuclear DNA compaction defect. Grey, DAPI. Scale bars, 10 µm. (**C**) Percentage of X- or Y-bearing spermatids among Ste-containing spermatids in males expressing the piRNA-resistant *Ste* transgene (β*Tub-Ste*$^{piRNA\text{-}resistant}$). The number of scored Ste-containing spermatids is shown above the bar. Statistical analysis was performed using two-sided Fisher's exact test (Null hypothesis: Ste-containing spermatids have equal chances of carrying X or Y chromosomes). ****$P < 0.0001$.

Fig. S6

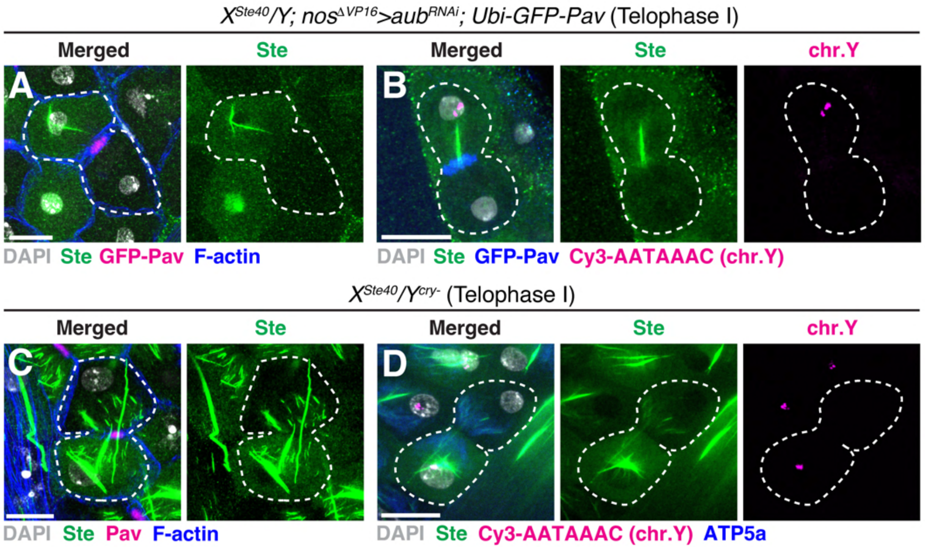

Fig. S6. Ste asymmetrically segregates to Y chromosome-bearing cells during meiosis I. (**A**) Immunofluorescence staining of a telophase I cell (indicated by white dotted lines) from $X^{Ste40}/Y; nos^{\Delta VP16}>aub^{RNAi}; Ubi\text{-}GFP\text{-}Pav$ male, exhibiting asymmetric Ste segregation. Ste (green), GFP-Pav (magenta), and F-actin (blue). Grey, DAPI. Scale bar, 10 µm. (**B**) Immunofluorescence and DNA-FISH staining of a telophase I cell (indicated by white dotted lines) from $X^{Ste40}/Y; nos^{\Delta VP16}>aub^{RNAi}; Ubi\text{-}GFP\text{-}Pav$ male, exhibiting asymmetric Ste segregation to the Y chromosome side. Ste (green), GFP-Pav (blue), and the Y chromosome (Cy3-AATAAAC, magenta). Grey, DAPI. Scale bar, 10 µm. (**C**) Immunofluorescence staining of a telophase I cell (indicated by white dotted lines) from X^{Ste40}/Y^{cry-} male, exhibiting asymmetric Ste segregation. Note that the relatively high expression of Ste in this genotype often resulted in both daughter cells inheriting Ste protein, although asymmetry remained apparent. Ste (green), Pav (magenta), and F-actin (blue). Grey, DAPI. Scale bar, 10 µm. (**D**) Immunofluorescence and DNA-FISH staining of a telophase I cell (indicated by white dotted lines) from X^{Ste40}/Y^{cry-} male, exhibiting asymmetric Ste segregation to the Y chromosome side. Ste (green), ATP5a (mitochondria decorating the spindle, blue), and the Y chromosome (Cy3-AATAAAC, magenta). Grey, DAPI. Scale bar, 10 µm.

Fig. S7

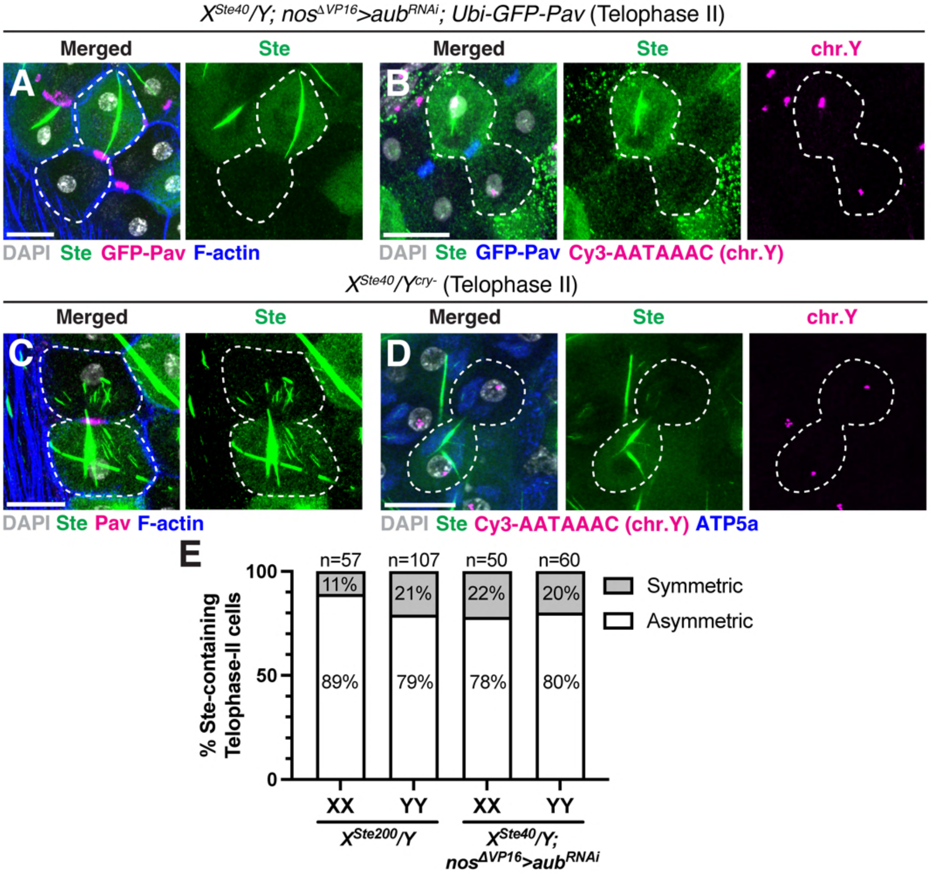

Fig. S7. Ste segregates asymmetrically during meiosis II. (**A**) Immunofluorescence staining of a telophase II cell (indicated by white dotted lines) from *X^Ste40^/Y; nos^ΔVP16^>aub^RNAi^; Ubi-GFP-Pav* male, exhibiting asymmetric Ste segregation. Ste (green), GFP-Pav (magenta), and F-actin (blue). Grey, DAPI. Scale bar, 10 µm. (**B**) Immunofluorescence and DNA-FISH staining of a telophase II cell (indicated by white dotted lines) from *X^Ste40^/Y; nos^ΔVP16^>aub^RNAi^; Ubi-GFP-Pav* male. Ste (green), GFP-Pav (blue), and the Y chromosome (Cy3-AATAAAC, magenta). Grey, DAPI. Scale bar, 10 µm. (**C**) Immunofluorescence staining of a telophase II cell (indicated by white dotted lines) from *X^Ste40^/Y^cry-^* male, exhibiting asymmetric Ste segregation. Ste (green), Pav (magenta), and F-actin (blue). Grey, DAPI. Scale bar: 10 µm. (**D**) Immunofluorescence and DNA-FISH staining of a telophase II cell (indicated by white dotted lines) from *X^Ste40^/Y^cry-^* male, exhibiting asymmetric Ste segregation. Ste (green), ATP5a (mitochondria decorating the spindle, blue), and the Y chromosome (Cy3-AATAAAC, magenta). Grey, DAPI. Scale bar, 10 µm. (**E**) Frequency of Ste asymmetry during meiosis II among Ste-containing cells in the indicated genotypes. The number of scored meiosis II cells is shown above the bars.

Fig. S8

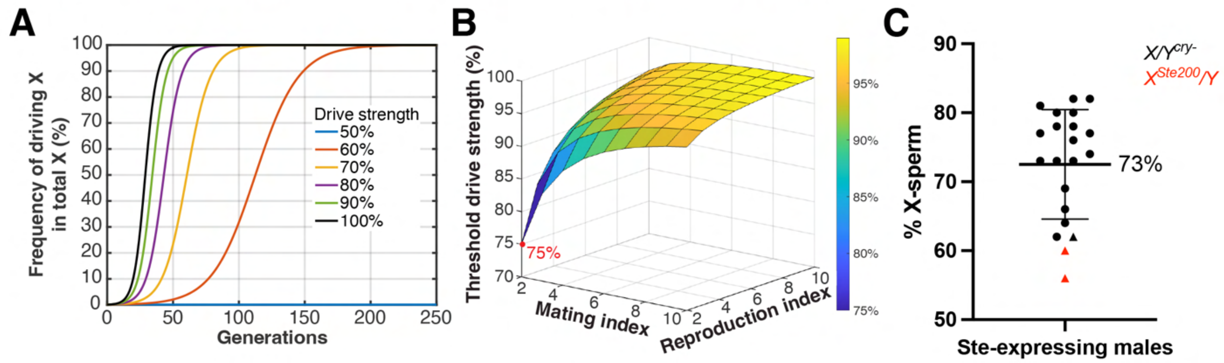

Fig. S8. *Ste*'s drive strength is likely below the threshold drive strength. (**A**) The frequency of the driving X chromosome in the total X chromosome pool of the population over generations, plotted against varying degrees of drive strength. (**B**) Threshold drive strength with varying mating index and reproduction index. 75% is the lowest threshold drive strength (see Materials and Methods). (**C**) Percentage of X chromosome-bearing sperm produced by Ste-expressing males of the indicated genotypes. Each dot in the graph represents a genotype. Data include results from our own study (triangular dots) and previous studies (circular dots) (*26*). Mean ± SD is indicated in the graph. The number of scored progenies for each genotype is shown in table S2.

Table S1. Different levels of Ste expression lead to distinct phenotypes.

Ste expression	Genotype	Phenotype
None	X^{Ste40}/Y	Wild type.
Moderate	X^{Ste200}/Y	• Ste localizes to a subset of spermatids, leading to the nuclear DNA compaction defect.<br>• Males are fertile.<br>• Males produce more females than males in the progeny.
	X^{Ste40}/Y; $nos^{\Delta VP16}>aub^{RNAi}$	
	X^{Ste40}/Y; $nos>aub^{RNAi}$	
	X^{Ste40}/Y; $bam>aub^{RNAi}$	
	X^{Ste40}/Y^{cry-}	
High	X^{Ste200}/Y; $bam>aub^{RNAi}$	• Ste causes the breakdown of entire spermatid cysts, resulting in no surviving sperm.<br>• Males are sterile.
	X^{Ste200}/Y^{cry-}	

Table S2. Percentage of X-sperm produced by Ste-expressing males across various genotypes.

Source	Genotype	Sperm genotype*		Sum	X:Y	X/(X+Y)
		X	Y			
This study	*X^{Ste40}/Y^{cry-}*	2924	1815	4739	1.6	62%
	X^{Ste200}/Y;bam-gal4	2646	2044	4690	1.3	56%
	X^{Ste200}/Y;Sp/CyO;TM2/TM6B	2475	1649	4124	1.5	60%
Palumbo et al., 1994 (*26*)	*W-12/Y^{cry-}*	1206	667	1873	1.8	64%
	Altamura-1/Y^{cry-}	1567	966	2533	1.6	62%
	Fairfield-11/Y^{cry-}	1261	354	1615	3.6	78%
	Salve-3/Y^{cry-}	1369	705	2074	1.9	66%
	Salve-2/Y^{cry-}	1720	374	2094	4.6	82%
	Altamura-46/Y^{cry-}	1219	343	1562	3.6	78%
	Altamura-66/Y^{cry-}	1645	576	2221	2.9	74%
	y w f/Y^{cry-}	1186	529	1715	2.2	69%
	Altamura-40/Y^{cry-}	1400	425	1825	3.3	77%
	Altamura-61/Y^{cry-}	1506	447	1953	3.4	77%
	Valenzano-2/Y^{cry-}	243	91	334	2.7	73%
	Sammichele/Y^{cry-}	233	57	290	4.1	80%
	Giovinazzo/Y^{cry-}	333	125	458	2.7	73%
	Altamura-4/Y^{cry-}	467	175	642	2.7	73%
	Altamura-22/Y^{cry-}	373	94	467	4.0	80%
	Salve-4/Y^{cry-}	120	29	149	4.1	81%
	Altamura-36/Y^{cry-}	184	41	225	4.5	82%
	Gandoli-6/Y^{cry-}	183	58	241	3.2	76%
Average	---				2.9	72.5%

* Non-disjunction products (XY- and O-sperm) are excluded in our analysis.

Table S3. cDNA sequence of piRNA-resistant *Ste*.

Gene	cDNA sequence (piRNA resistant)
Ste	ATGAGCTCCTCGCAGAATAATAATTCCTCCTGGATTGACTGGTTCTT<br>GGGCATTAAGGGAAATCAATTCTTGTGCAGGGTGCCGACGGATTAC<br>GTCCAAGACACCTTTAATCAAATGGGACTGGAATACTTTTCCGAAA<br>TCTTGGATGTCATTCTCAAACCCGTGATTGATTCCAGCAGCGGACTG<br>CTCTATGGAGACGAGAAGAAATGGTATGGAATGATCCATGCTAGGT<br>ATATTCGCAGCGAAAGGGGATTGATCGCCATGCATAGGAAGTACAT<br>GAGGGGCGACTTCGGCAGCTGCCCGAACATTAGCTGCGACCGCCAA<br>AATACGCTGCCCGTGGGATTGTCCGCCGTGTGGGGAAAGAGCACGG<br>TGAAAATTCATTGTCCGAGATGCAAGTCCAATTTCCACCCCAAGAG<br>CGACACCCAACTGGATGGCGCCATGTTTGGACCGTCCTTCCCCGAT<br>ATTTTTTTCAGCATGTTGCCAAATCTGACCAGTCCGCTGGATGATCC<br>GAGGACCTAA

Table S4. Fertility assay for males expressing different levels of Ste.

**Ste expression**	**No**	**Moderate**		**High**
**Genotype**	X^{Ste40}/Y	X^{Ste200}/Y	X^{Ste40}/Y^{cry-}	X^{Ste200}/Y^{cry-}
male 1	105	75	125	0
male 2	89	80	113	0
male 3	92	76	110	0
male 4	94	115	64	0
male 5	103	85	89	0
male 6	106	102	109	0
male 7	93	75	86	0
male 8	85	85	114	0
male 9	58	69	103	0
male 10	112	109	102	0

Table S5. The distorted sex ratio in Ste-expressing males is rescued by Ste RNAi

Genotype	*X^{Ste40}/Y; bam-gal4*			*X^{Ste200}/Y; bam-gal4*			*X^{Ste200}/Y; bam>ste^{RNAi}*		
**Individual**	**Female**	**Male**	**Female/Male**	**Female**	**Male**	**Female/Male**	**Female**	**Male**	**Female/Male**
male 1	284	264	1.08	301	220	1.37	282	243	1.16
male 2	216	221	0.98	281	216	1.30	260	244	1.07
male 3	278	277	1.00	289	260	1.11	292	240	1.22
male 4	254	265	0.96	338	227	1.49	302	258	1.17
male 5	249	226	1.10	264	238	1.11	269	282	0.95
male 6	222	295	0.75	283	262	1.08	288	253	1.14
male 7	285	265	1.08	196	121	1.62	173	169	1.02
male 8	164	192	0.85	185	167	1.11	160	161	0.99
male 9	111	119	0.93	150	104	1.44	206	188	1.10
male 10	242	273	0.89	359	229	1.57	296	239	1.24

Table S6. Quantification of Ste-containing spermatids.

Genotype	Ste-containing spermatids				Y/X+Y (%)	Non-disjunction (%)
	X	Y	XY or O	Total		
X^{Ste200}/Y	70	507	13	590	88%	2%
X^{Ste40}/Y; nos^{DVP16}>aub^{RNAi}	82	197	11	290	71%	4%
X^{Ste40}/Y; nos>aub^{RNAi}	56	156	1	213	74%	0%
X^{Ste40}/Y; bam>aub^{RNAi}	71	168	6	245	70%	2%
βTub-$Ste^{piRNA\text{-}resistant}$/Y	13	93	0	106	88%	0%

Table S7. Fisher's exact test results for Ste's preferential localization in spermatids.

Genotype		Ste-containing spermatids			*P* value	Asterisks
		**X**	**Y**	**Total**		
X^{Ste200}/Y	Expected	288	289	577	<0.0001	****
	Observed	70	507	577		
$X^{Ste40}/Y; nos^{DVP16}>aub^{RNAi}$	Expected	139	140	279	<0.0001	****
	Observed	82	197	279		
$X^{Ste40}/Y; nos>aub^{RNAi}$	Expected	106	106	212	<0.0001	****
	Observed	56	156	212		
$X^{Ste40}/Y; bam>aub^{RNAi}$	Expected	119	120	239	<0.0001	****
	Observed	71	168	239		
$bTub\text{-}Ste^{piRNA\text{-}resistant}/Y$	Expected	53	53	106	<0.0001	****
	Observed	13	93	106		

Null hypothesis: Ste-containing spermatids have equal probabilities of containing either X or Y chromosomes.

Table S8. Fisher's exact test results for the association of Ste with the Y chromosome in meiosis I.

**Genotype**		**Ste-containing spermatids**			***P* value**	**Asterisks**
		**X**	**Y**	**Total**		
X^{Ste200}/Y	Expected	56	56	112	<0.0001	****
	Observed	20	92	112		
X^{Ste40}/Y; nos^{DVP16}>aub^{RNAi}	Expected	93	94	187	0.0121	*
	Observed	68	119	187		
X^{Ste40}/Y^{cry-}	Expected	101	102	203	0.0018	**
	Observed	69	134	203		

Null hypothesis: Ste has equal probabilities of segregating with the X or Y chromosome during meiosis I.

MATLAB code for the mathematical modeling
(also available at https://github.com/xuefengmeng/Meng_et_al_2024.git)
Fig4A.m
Fig4B.m
Fig4C.m
Fig4D.m
Fig4E.m
FigS8A.m
FigS8B.m
are used to obtain the modeling results presented in Figure 4 and Figure S8.
